# Two Cases of Acute Abdominal Disease

**Published:** 1908-05-30

**Authors:** 


### CASES FOR DIAGNOSIS.

### TWO CASES OF ACUTE ABDOMINAL DISEASE.

THE difficulty of diagnosing the causes of acute abdominal conditions is often so great that one has to be content, in many instances, with deciding which of two groups an individual case should be put into, namely:—

(1) Acute abdominal conditions requiring immediate laparotomy.

(2) Acute abdominal conditions *not* requiring immediate laparotomy.

It requires the greatest care, even to be successful in relegating cases to one or other of these broad groups, and fortunate is the physician who makes fewest mistakes. Moreover, it is comparatively easy to conclude that the trouble is abdominal when the real lesion is in the chest, or even in the head.

When laparotomy has been decided upon it is more than likely that one or other of the commoner causes will be found, particularly appendicitis, perforated gastric or duodenal ulcer, intestinal obstruction, pelvic abscess, ruptured ectopic gestation, or ovarian cyst with twisted pedicle. On the other hand the condition may be something much less usual. The two following cases are described here up to the time when the line of treatment to be adopted was decided in consultation. The treatment acually adopted and the nature of the lesion will be given in our next issue. The questions that had to be answered as best they could be from the facts before the doctors at the time were:—

(1) What treatment is most likely to save the patient's life?

(2) What is the probable diagnosis of the case?

(3) Are there any other clinical means we might adopt to help us to arrive at the correct diagnosis with greater certainty.

*Case* 1.—A married woman, aged 59, was in perfect health until June 10; the menopause came on naturally at 47; she lived a regular and temperate life, was the mother of 10 living children, and of three others who had died; had had no miscarriages. She was strongly built, accustomed to work at the wash-tub, and had not been ill since she could remember, except for her confinements. On June 10, without any apparent cause, she suddenly had a severe rigor; within an hour she had acute pain in the lower part of her abdomen, and in her pelvic region; she vomited repeatedly, and was helped to bed by her friends. She stayed in bed, was unable to eat anything without vomiting at once, and continued to have acute pain in her abdomen. The bowels had been open on the 10th, but had since been confined, and were still so on June 13, when a consultation was held. By this time the vomiting had become fæculent, the patient's temperature was 102° F., her pulse-rate 120, and her respiration-rate 26 per minute. No abnormal physical signs could be detected in her lungs or heart. The knee-jerks were present, and the eyes reacted to light and to accommodation. There was no blue line upon the gums. The conjunctivæ were tinged pale yellow, though there was no obvious jaundice elsewhere. The urine contained a small quantity of bile pigments—a trace of albumen without casts, and much indican, but otherwise was natural. Rectal and vaginal examinations were both negative, the rectum being empty, and the fornices of the vagina natural. There was no hernia to be noticed in the femoral, inguinal, or umbilical regions. The patient lay flat upon her back in bed with her knees drawn up; her face was expressive of pain, but was not sunken about the eyes or elsewhere. The tongue was coated with a white fur, but was fairly moist. The abdomen was uniformly distended and did not move on respiration, but it was perfectly supple and not rigid anywhere. There was some tenderness on palpation in the epigastric region, but there was altogether much less tenderness than pain. There was a slightly impaired note in both flanks, but the dullness did not move when the patient shifted from side to side. The liver dullness was present and natural; neither liver nor spleen could be felt. There was no visible peristalsis. No peritonitic rub could be heard over liver, spleen, or elsewhere. There was good pulsation in both femoral arteries.

What was the matter? What was done? What happened?

*Case* 2.—A married woman, aged 47, was seen on June 3, when she was suffering from very acute abdominal pain. The history was that she had hitherto regarded herself as strong and well. She had had no serious illness before, though she had sometimes had temporary twinges of pain in her abdomen, particularly on the right side in the lower part. She had paid no particular attention to these, however, for they had never been severe enough to make her lie up in bed. She had a large family, the youngest child being some years old; the menstrual periods had been quite regular, and she was expecting one about the date of her attack. Having been out-of-sorts, without any very definite symptoms, for three or four days, she was suddenly seized with acute abdominal pain early in the morning of June 3. The pain was worst in the right iliac fossa to begin with, and she vomited several times; in a few hours the pain was generalised all over the abdomen, so that she could not say particularly where it was. Upon examination the patient was found to be lying upon her back with her knees drawn up, her face drawn, her eyes a little sunken, and her tongue brown and dry. The teeth were carious and dirty; there was no blue line upon the gum. The knee-jerks were present, and the pupils reacted to light and to accommodation. The heart and lungs seemed natural. The temperature was 103° F., pulse-rate 100, respiration-rate 24. Rectal examination and vaginal examination showed that there was acute sensitiveness to pressure upwards and to the right, but no lump nor fullness could be felt there. There were fæces in the rectum, though the bowels had been opened since the abdominal pain began. The urine was found to be of normal specific gravity, but it contained a small though definite amount of blood, and a small amount of albumen; it was thought at the time that the blood might be due to the onset of the expected menses. Microscopical examination showed red corpuscles and an excess of leucocytes, but no crystals and no renal-tube casts. The optic discs and retinæ were natural. The abdomen was well covered with fat, and difficult to palpate properly; it was uniformly distended, moved extremely little with respiration, and was rigid all over; the rigidity was perhaps slightly more marked over the right iliac fossa than elsewhere, and there was rather more fullness here than in other places. The patient had no great tenderness on light palpation of the abdomen, but on anything like firm pressure over the right iliac fossa she suffered greatly. The vomiting was persistent, the material brought up being bile-stained, but quite free from blood. The liver dullness was diminished, extending only from the fifth right space to the seventh rib in the right nipple line; neither liver nor spleen could be felt; no peritonitic rub could be made out, and there was no dullness in the flanks. There was normal pulsation in the arteries of the legs, and no œdema anywhere. An enema was given, with a good result, but the pulse-rate rose to 115 in an hour's time and the vomiting and retching were very severe. There was no jaundice.

What was the matter? What was done? What happened?

[The solutions of these two cases will appear in our next issue.—Ed.]

